# Signalling and responding to zoonotic threats using a One Health approach: a decade of the Zoonoses Structure in the Netherlands, 2011 to 2021

**DOI:** 10.2807/1560-7917.ES.2022.27.31.2200039

**Published:** 2022-08-04

**Authors:** Joke van der Giessen, Frits Vlaanderen, Titia Kortbeek, Corien Swaan, Hans van den Kerkhof, Els Broens, Jolianne Rijks, Miriam Koene, Mauro De Rosa, Mathilde Uiterwijk, Marieke Augustijn-Schretlen, Catharina Maassen

**Affiliations:** 1 Centre of Infectious Disease Control of the National Institute for Public Health and the Environment (Cib-RIVM), Bilthoven, The Netherlands; 2 Faculty of Veterinary Medicine, Utrecht University, Utrecht, The Netherlands; 3 Dutch Wildlife Health Centre (DWHC), Utrecht University, Utrecht, The Netherlands; 4 Wageningen Bioveterinary Research (WBVR), Lelystad, The Netherlands; 5 Netherlands Food and Consumer Product Safety Authority (NVWA), Utrecht, The Netherlands; 6 Centre for Monitoring of Vectors (CMV), Netherlands Institute for Vectors, Invasive plants and Plant health (NIVIP), Netherlands Food and Consumer Product Safety Authority (NVWA), Wageningen, the Netherlands; 7 Royal GD, Deventer, The Netherlands

**Keywords:** 10 years experience, response to emerging zoonoses, Zoonoses Structure, One Health, One Health risk analysis system

## Abstract

In the Netherlands, the avian influenza outbreak in poultry in 2003 and the Q fever outbreak in dairy goats between 2007 and 2010 had severe consequences for public health. These outbreaks led to the establishment of an integrated human-veterinary risk analysis system for zoonoses, the Zoonoses Structure. The aim of the Zoonoses Structure is to signal, assess and control emerging zoonoses that may pose a risk to animal and/or human health in an integrated One Health approach. The Signalling Forum Zoonoses (SO-Z), the first step of the Zoonoses Structure, is a multidisciplinary committee composed of experts from the medical, veterinary, entomology and wildlife domains. The SO-Z shares relevant signals with professionals and has monthly meetings. Over the past 10 years (June 2011 to December 2021), 390 different signals of various zoonotic pathogens in animal reservoirs and humans have been assessed. Here, we describe the Zoonoses Structure with examples from signals and responses for four zoonotic events in the Netherlands (tularaemia, *Brucella canis*, West Nile virus, and severe acute respiratory syndrome coronavirus 2 (SARS-CoV-2)). This may serve as an example for other countries on how to collaborate in a One Health approach to signal and control emerging zoonoses.

## Background

As a densely populated country, with very high numbers of livestock and poultry, and close presence of wildlife, the Netherlands are vulnerable to the emergence of zoonoses [1]. The first decade of this century saw three outbreaks of emerging zoonoses: the avian influenza outbreak in 2003, livestock-associated methicillin-resistant Staphylococcus aureus in 2007 and the largest Q fever outbreak ever reported in 2007–2010 [2]. In 2009, as a result of the national Emerging Zoonoses programme [3] a pilot group of medical and veterinary experts from public health (National Institute for Public Health and the Environment, RIVM), animal health (Wageningen Bioveterinary Research, WBVR, and Royal GD) and the Netherlands Food and Consumer Product Safety Authority (NVWA) was formed. The aim of the pilot group was to build a blueprint for a systematic approach of sharing and assessing signals of emerging zoonotic pathogens in humans and animals between veterinary and medical professionals. As the formal evaluation of the Q fever epidemic [4], showed a clear need for such an integrated approach, this pilot group was formalised as the national Signalling Forum for Zoonoses (SO-Z). The SO-Z is the first step of the national Zoonoses Structure, an integrated human-veterinary risk analysis system formally established in 2011 by the Dutch Ministry of Health, Welfare and Sport and the Ministry of Economic Affairs, Agriculture and Innovation (the current Ministry of Agriculture, Nature and Food Quality). The aim of the Zoonoses Structure is to signal, assess and control (potentially) emerging zoonoses that may pose a risk to animal and/or human health, in an integrated One Health approach. Here we describe the Zoonoses Structure with examples from signalling to responses for four zoonotic events in the Netherlands.

## Set up of the Zoonoses Structure

The Zoonoses Structure follows the infectious diseases control system in the Netherlands, see Figure for structure and organisations involved.

The SO-Z is a multidisciplinary committee responsible for sharing and assessing signals of emerging zoonotic pathogens and informing the necessary parties within the Zoonoses Structure (Figure). SO-Z is composed of 18 experts from the medical, veterinary, entomology and wildlife domains. Members (and substitutes) are appointed by different institutes, i.e. Royal GD, Faculty of Veterinary Medicine, Dutch Wildlife Health Centre, WBVR, the Incidence Crisis Centre at NVWA and Dutch National Centre for Monitoring of Vectors at NVWA, and RIVM. Ad hoc experts are consulted when needed. The legal backbone of the SO-Z is based on a collaboration agreement. Changes to the collaboration agreement require approval from both the Ministry of Health, Welfare and Sports and the Ministry of Agriculture, Nature and Food Quality. The SO-Z collaboration agreement describes the role and expertise of the representatives, the role, tasks and origin of the appointed chair and secretary, how signals are collected, the frequency and location of meetings and how reporting and financing are organised.

**Figure:**
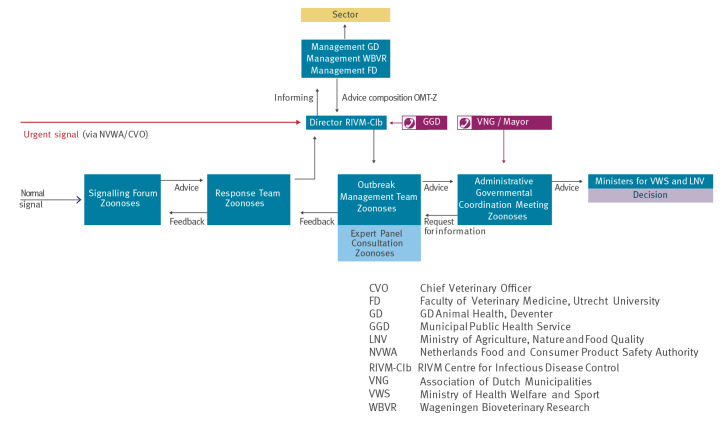
Schematic overview of The Zoonoses Structure containing SO-Z; RT-Z; OMT-Z; AGCM-Z; EPC-Z)

AGCM-Z (Administrative Governmental Coordination Meeting Zoonoses): administrative organisations involved in the control of the outbreak judge advised measurements of the OMT-Z on governmental feasibility and desirability. Conclusively, decision-making on control measures takes place at the governmental level.

EPC-Z (Expert Panel Consultation Zoonoses): an expert consultation can be organised in less urgent cases, which extensively analyses existing evidence and knowledge gaps. Recommendations are made about control strategy as well as on research strategy in order to obtain the relevant knowledge for risk assessment and/ or control measures.

OMT-Z (Outbreak Management Team Zoonoses): formed in case of an outbreak for which guidelines on outbreak control do not exist, or do not cover the specific outbreak situation. Experts assess the signal in depth and advice the AGCM-Z about the risk and appropriate control measures.

RT-Z (Response Team Zoonoses): response including upscaling. Full assessment of signal and advice on strategy to control spreading, possible interventions, diagnostics and treatment, communication.

SO-Z: Signalling Forum Zoonoses. Signalling and first assessment of (potentially) zoonotic infections.

This structure for the control of zoonoses is based on the existing Dutch structure for the control of infectious diseases.

Source: Ministry of Health, Welfare and Sport, Ministry of Agriculture, Nature and Food Quality (update 2018, by RIVM).

The chair of the SO-Z informs the chair of the RT-Z/EPC-Z (head of the National coordination Centre for Infectious Disease Control at RIVM) in case a signal needs further action. Signals are reported to the chair of RT-Z when the following criteria are met: (i) when outbreaks of known zoonoses occur with higher frequency or severity; (ii) when outbreaks of zoonoses occur that cannot be treated (easily); (iii) in case of a new or emerging zoonosis and (iv) in the case of social unrest. As defined in the zoonoses structure, the director of the Centre for Infectious Disease Control (RIVM-Cib) is the chair of the OMT-Z.

The SO-Z meets monthly to confidentially share, discuss, and assess signals in human and animal health. In the event of a (potentially) urgent threat, ad hoc assessments are organised. If an assessment identifies a potential public health threat, the chair of the Response Team Zoonoses (RT-Z) is informed by the chair of the SO-Z, based on at least one of the following criteria as stated in the collaboration agreement: (i) when outbreaks of known zoonoses occur with higher frequency or severity; (ii) when outbreaks of zoonoses occur that cannot be treated (easily); (iii) in the case of a new or emerging zoonosis; (iv) in the case of social unrest.

Based on risk assessment directed by the SO-Z and/or advice by the chair of the RT-Z/EPC/Z, normally in case of quickly developing crisis for which no or insufficient guidelines exist, the Outbreak Management Team Zoonoses (OMT-Z) will be convened by the director of the Centre for Infectious Disease Control (RIVM-Cib), to advise the Minister of Health, Welfare and Sports and the Minister of Agriculture, Nature and Food Quality. The chairs and secretariats of all steps in the Zoonoses Structure are appointed by both Ministries and are parallel to the Dutch public health structure (Figure).

The SO-Z shares relevant signals with veterinary and medical professionals and others working in the field of zoonoses via email. Important signals are actively shared via newsletters with veterinary (named Vetinf@ct) and/or public health (named Labinf@ct or Inf@ct) professionals.

## Signals followed up in the past 10 years

From June 2011 to December 2021, 390 different signals of various zoonotic pathogens in animal reservoirs and humans were assessed. Four zoonotic signals are described here in more detail in terms of their origin (wildlife, imported dogs, livestock, vector-borne) and the follow up actions taken in the Zoonoses Structure.

### Tularaemia

After an apparent absence for several decades, the first autochthonous tularaemia case in a human patient was reported in the Netherlands in 2011, followed by a finding in a European brown hare (*Lepus europaeus*) in 2013 [5,6]. Thereafter, autochthonous cases have been identified regularly, with a total of 26 human and 54 wildlife cases to date (December 2021). After the initial tularaemia cases were reported, the SO-Z together with a multidisciplinary tularaemia working group closely tracked human and animal cases and performed risk assessments. Information was provided through websites and through SO-Z signals in monthly newsletters (n = 16) to One Health professionals, to veterinary professionals as Vetinf@ct (n = 3), and to medical microbiologists as Labinf@cts and Inf@cts (n = 2). Regular updates covered information on clinical manifestations or transmission routes in humans and wildlife, geographical distributions and genetic diversity of Dutch *Francisella* isolates. In 2015, tularaemia caused mass mortality in hares in a localised outbreak, increasing sense of urgency, where SO-Z called for a scale up, informing the Response Team Zoonoses (RT-Z). The RT-Z decided on swift communication to local risk groups and professionals at field level, and on a full environmental (animal-vector-water) investigation in the outbreak area. In 2016, notification for humans was made mandatory. No human cases were found to be associated with the 2015 tularaemia outbreak in hares [7].

### Brucella canis

*Brucella canis* had not been detected in the Netherlands until November 2016, when it was isolated from a dog with discospondylitis. The case was reported to the SO-Z and tracing investigations by the Incidence Crisis Centre of the NVWA revealed that the dog had been imported from Romania 9 months prior to the diagnosis [8]. Multiple notifications from veterinary professionals followed the publication of a Vetinf@ct. Track and trace investigations discovered 18 canine cases in the following 2 years. Dogs testing positive for *B. canis* had been imported from Eastern Europe or Russia on average 11 months (range 2–32 months) before clinical signs were seen. The chair of the SO-Z informed the chair of the Expert Panel Consultation Zoonoses (EPC-Z) and in December 2017, an EPC-Z was organised by CIb-RIVM to assess the risks for human and animal health. Advice from the EPC-Z was to explore the possibilities for (mandatory) control measures for pets, to send a Labinf@ct to medical doctors and medical laboratories about the lack of accurate tests for *B. canis* in humans, and to perform a seroprevalence study among imported dogs [9].

### West Nile virus

West Nile virus (WNV) occurs in the endemic regions of southern Europe mostly in the months of July to November, and is consistent with seasonal amplification of WNV in mosquitos and birds [10]. In 2018, the first autochthonous WNV cases were found in birds, horses, and a person in Germany [10,11]. Because WNV was found in our neighbouring country, these signals were closely followed in the SO-Z meetings between September and December 2018 and shared with the professional network via monthly emails. In August 2020, WNV was detected in a migratory bird and in two pools of mosquitoes caught in Utrecht municipality in the Netherlands [12]. The Zoonoses Structure was scaled up to a RT-Z. The RT-Z recommended three measures: (i) intensification and expansion of WNV surveillance in mosquitoes, birds, and horses in the area where the virus was detected; (ii) sera surveillance among blood donors; (iii) (retrospective) screening of patients with neurological disease of unknown aetiology. In October 2020, the first autochthonous human WNV infection detected in the Netherlands was later followed later by the detection of seven more human cases, all residing in the central part of the Netherlands (around Utrecht and Arnhem municipalities), presumably infected in July–September 2020 [13].

### Severe acute respiratory syndrome coronavirus 2 in minks

Severe acute respiratory syndrome coronavirus 2 (SARS-CoV-2) was introduced by infected humans to farmed mink in the Netherlands in 2020 [14,15]. After the first reports of SARS-CoV-2 in minks in May 2020, the Ministry of Agriculture, Nature and Food Quality, imposed a notification obligation with immediate effect, as the minks could potentially spread the virus in the affected area. In addition to the notification obligation, other measures taken included: (i) a ban on the transport of minks and their manure; (ii) a stricter hygiene protocol; (iii) a visitor ban on all mink farms. In addition, dogs and cats living on a mink farm needed to be kept on the farm. Minks at all farms in the Netherlands were screened for SARS-CoV-2 antibodies, and an early warning system was set up in which minks that died naturally were sent to Royal GD weekly and examined for SARS-CoV-2 by means of PCR. Veterinarians were informed via several Vetinf@cts about the control of SARS-CoV-2 in minks and the measures needing to be taken for dogs and cats [16]. Despite all measures, farm-to-farm transmission did not stop. Based on the advice of the first OMT-Z [17,18] and the Administrative Governmental Coordination Meeting Zoonoses (AGCM-Z), the Ministries of Health, Welfare and Sport and of Agriculture, Nature and Food Quality, decided on 3 June 2020 that all minks at infected farms had to be culled. The OMT-Z advice was based on extensive investigations carried out on the first infected farms. On 28 August 2020, the Dutch government decided that all mink farms had to stop production before the next season based upon advice given by OMT-Z after further restrictive measures did not stop the spread of the virus on mink farms. Since 8 January 2021, it is prohibited to keep mink for fur production and all Dutch mink farms were closed [19].

## Lessons learned from the risk analysis system for zoonoses

The emergence of infectious diseases from animal reservoirs shows that sustainable solutions in a One Health approach, involving interaction between medical, veterinary, and environmental domains, are necessary to protect public and animal health. Using the Zoonoses Structure in the Netherlands, it was possible to share and respond to confidential signals between sectors more efficiently. Moreover, we experienced that permanent representatives and substitutes in the SO-Z favoured building trust between experts. The sense of urgency of signals, particularly signals occurring only in the animal reservoir without human cases, can differ between experts in different domains. This was the case in the example of *Brucella canis* found in dogs. Veterinary experts urged for immediate follow-up and strict measures to limit the import of *B. canis*-positive dogs in order to prevent human exposure. However, experts from the human domain were less alarmed as no human cases had been observed and the risk to the general public was assessed as low. Better defined criteria or a One Health rapid risk assessment tool could possibly help assess the signals between domains with different perspectives.

Since the start of Zoonoses Structure in 2011, the collaboration agreement was updated once to clarify certain sections, giving more detail. Describing the collaboration agreement in precise detail is important when there is collaboration between partners of different domains.

The two formalised zoonoses signalling collaborations that currently exist in Europe, Human Animal Infections and Risk Surveillance Group (HAIRS) in the United Kingdom [20] and Zoonoses Structure in the Netherlands, were set up after a zoonotic disease crisis (bovine spongiform encephalopathy and Q fever, respectively). For such a One Health collaboration to work successfully, building of trust is a time consuming but essential component. Therefore, it is advisable to build such systems in peacetime, so they are ready when a crisis occurs.

There is no blueprint on how to organise One Health risk analysis systems, since every country is organised differently and has different geographic, cultural, and governmental challenges. The One Health European Joint Project COHESIVE has developed a web-based implementation guideline to support countries in setting up an operational One Health risk analysis system [21]. In addition, the Tripartite (the World Organization for Animal Health, the World Health Organization and the Food and Agriculture Organization) has published the ‘Tripartite Guide to Addressing Zoonotic Diseases in Countries’, and is currently working to support implementation of One Health surveillance in more countries [22].

## Conclusion

The Zoonoses Structure in the Netherlands has shown to be of added value in the early warning and control of new and emerging zoonoses. There are sustainable relationships and trust between professionals in the involved domains facilitating the exchange of necessary information in order to tackle upcoming emerging zoonotic threats. A clear description of tasks and responsibilities, as well as governmental support, are crucial factors.
